# Intra-household Variation in Pathways to Care for Epilepsy and Mental Disorders in Eastern Uganda

**DOI:** 10.3389/fpubh.2021.583667

**Published:** 2021-07-26

**Authors:** Nandini D. P. Sarkar, Azucena Bardaji, Florence K. Baingana, Joan Muela Rivera, Bart Criel, Joske Bunders-Aelen, Koen Peeters Grietens

**Affiliations:** ^1^Department of Public Health, Institute of Tropical Medicine, Antwerp, Belgium; ^2^Athena Institute for Research on Innovation and Communication in Health and Life Sciences, Faculty of Sciences, Vrije Universiteit Amsterdam, Amsterdam, Netherlands; ^3^ISGlobal, Hospital Clinic – University of Barcelona, Barcelona, Spain; ^4^School of Public Health, College of Health Sciences, Makerere University, Kampala, Uganda; ^5^PASS Suisse, Neuchatel, Switzerland; ^6^Medical Anthropology Research Centre (MARC) at Departament d'Antropologia, Filosofia i Treball Social, Universidad Rovira i Virgili, Tarragona, Spain

**Keywords:** Uganda, low middle income country, qualitative research, epilepsy, mental health, pathways to care, help-seeking behavior, caregivers

## Abstract

Integrating mental, neurological, and substance use (MNS) health care into the public health system has become a global priority, with mental health, and well-being now being part of the Sustainable Development Goals. In the aim to provide good quality care for MNS disorders, understanding patients' pathways to care is key. This qualitative study explores the pathways to care of patients attending an outpatient mental health clinic of a district hospital in eastern rural Uganda, from the perspectives of their caregivers. Twenty seven in-depth interviews were conducted with caregivers of MNS patients visiting the clinic, with a focus on four case-presentations. Data analysis consisted of thematic and emergent content analyses using NVivo 11. Results across all interviews highlight that chosen help-seeking itineraries were largely pluralistic, combining and alternating between traditional healing practices, and biomedical care, regardless of the specific MNS disorder. Intra-household differences in care seeking pathways—e.g., where one patient received traditional help or no care at all, while the other received biomedical care—depended on caregivers' perceived contextual illness narrative for each patient, in combination with a variety of other factors. If interpreted as a form of bewitchment, traditional medicine and healing was often the first form of care sought, while the mental health clinic was seen as a recourse to “free” care. Patients, especially younger children, who showed visible improvements once stabilized on psychotropic medication was a source of motivation for caregivers to continue with biomedical care at the mental health clinic. However, stock-outs of the free psychotropic medication at the clinic led to dissatisfaction with services due to out-of-pocket expenses and precipitated returning to alternative therapy choices. This article showcases the importance of understanding the complex and varied combinations of individual, cultural, socioeconomic and structural factors that may affect caregivers' choices of pathways to care for patients with MNS disorders in eastern rural Uganda. These cumulative complex processes and context-specific help-seeking behaviors, which ultimately impact patient treatment and MNS health outcomes, need to be first acknowledged, understood and taken into account if we are to promote more inclusive, effective and integrated public mental health systems globally.

## Introduction

“*It used to work like demons. She became ill and [had] headaches and walked aimlessly, like demons… She would fall down and do inappropriate things… we would take her to the traditional healers … we tried the medical doctors …then we decided to use those tablets… and whenever she swallowed them, she couldn't fall down*.”—Caregiver SN023.

Mental, neurological, and substance use (MNS) disorders are among the leading causes of long-term disability and debilitation globally, accounting for 7.4–13% of disability-adjusted life-years (1, 2). On average, ~1 in 5 people experience a common mental disorder in any given 12 month period (3), while current estimates indicate that 14.3% of deaths are related to mental disorders globally (4). Epilepsy, a neurological disorder, had a global age-standardized prevalence of 621.5/100,000 population in 2016, with the highest prevalence in eastern, western, and southern sub-Saharan Africa regions, central Asia, central and Andean Latin America, and southeast Asia (5). As such epilepsy is considered a major public health problem in sub-Saharan Africa, and ~60% of persons living with epilepsy do not receive biomedical anti-epilepsy treatment (6). While low- and middle-income countries carry a significant amount of the disease burden from MNS disorders, their resource-constrained mental health systems continue to face large treatment gaps and shortages (1, 7).

Uganda, a low-income country in East Africa, has taken significant steps toward developing a national mental health policy in the last decade (8–10). Nevertheless, significant challenges remain in ensuring adequate integration of biomedical MNS health care into the decentralized Ugandan primary health care system (11). Human and financial resource constraints are primary barriers in ensuring equitable coverage at the local health system level (12). In general, the coverage, access to and quality of MNS services remains low, and are in part associated to socio-cultural and systemic barriers (13, 14).

Additionally, alternative systems of care may compete with the limited biomedical care available for MNS disorders through the public health system within these contexts. It has been hard to estimate the exact numbers of traditional healers in the Ugandan context, despite the government urging their registration and indexation (15). There is also a known vast diversity in traditional providers ranging from herbalists to diviners, faith healers, spirit mediums, and so on. As such, traditional care and its utilization has been documented for a wide variety of biomedical and psychosocial health concerns, including but not limited to HIV, malaria, diabetes, perinatal depression, spirit possession, and severe mental illness (16–22).

Regardless of the pathways of care of those living with MNS disorders, it is important to recognize that persons with MNS disorders require some degree of caregiving. While it is known that MNS disorders often extol a significant burden and associated cost on both patients and their caregivers (23, 24), there is limited data on caregiver perceptions toward the illness experience, their motivations, and choice of therapeutic itineraries. The few studies on caregivers in Uganda, focus on caregivers of children living with HIV/AIDS, and how caregivers can be supported (25–27); or the lived experiences of caregivers with patients living with MNS disorders and how specific interventions can support them (28, 29). Only one recent study specifically explores help-seeking processes amongst the parents of children with MNS disorders, which highlights a mixture of traditional, environmental, and biomedical understandings as underlying causes of illness that influence chosen pathways to care (30).

As such, there is value in evaluating MNS needs from caregiver perspectives. Theoretical models for conceptualizing and understanding health seeking behavior—, such as the Health Belief Model (31, 32), the PASS-model (33), and other anthropological and socio-cognitive models—can be useful tools in this regard. While normally used to situate patient perspectives, these frameworks can also be employed to understand and evaluate MNS-related service utilization, not only from the perspectives of service users, but their caregivers as well (34).

Additionally, in contexts where decision-making processes are highly dependent on the culture and surrounding social system, it would be highly relevant to take into account the role of the therapy management group (that is, those individuals who perform and partake in joint caregiving responsibilities of a patient), social support systems, social values, and social pressures (35). These factors, in combination with barriers in quality of, and access to, care and resource seeking, create deeply intertwined decision-making spaces that can be relevant to caregiver experiences, and coping strategies. To improve access to and utilization of publicly available MNS care in resource-constrained settings, it can therein be crucial to understand the decision-making processes of caregivers of persons with MNS disorders.

Thus, the aims of this study were: (a) to explore beliefs and attitudes of caregivers of persons with MNS disorders regarding their patient's particular MNS concern; (b) to understand the role of socio-cultural and socio-economic factors in caregiver decision-making; and (c) to assess caregivers' perceived quality of, and satisfaction with, available biomedical (whether pharmacological or psychological) treatment, and other available alternative pathways to care in eastern rural Uganda.

## Methods

### Study Design

This article presents data from a qualitative strand of research in a larger study conducted from September 2016 to February 2017. The larger study consisted of a mixed-methods assessment of Iganga district hospital's mental health clinic (MHC) and its visiting patients and caregivers, from a quality of care perspective (Author, submitted article). This qualitative enquiry focuses on the mechanisms underlying the help-seeking related, decision-making processes of caregivers of persons with MNS disorders visiting the MHC.

### Study Site and Population

The study took place across the rural district of Iganga in eastern Uganda, which is located in the Busoga sub-region. The district had an estimated total population of 504,197 inhabitants in 2014 with ~58% of the total population under the age of 18 (36). The district can be considered representative of most Ugandan rural populations, with subsistence farming being the main source of earning and livelihood of over 60% of Busoga households (36). The primary languages spoken in the district are Lusoga, Luganda, English and Swahili, while religious affiliations include Protestants, Muslims, Catholics, faith-based, and traditional cultural beliefs (37).

The local health system in Iganga district consists of four levels of care and is illustrative of the decentralized public health system in Uganda, providing free of cost biomedical care. Iganga district has one district level general hospital in Iganga town, which is a semi-urban township. Iganga district hospital serves a catchment population of ~1.5 million people, coming from across six neighboring districts. Within the district hospital there is the outpatient MHC, which is the first point of access to outpatient neuropsychiatric care at the district level.

According to hospital records, in the 12 month preceding the study period, the MHC had 4,613 patient contacts in total, amounting to a median of 355.5 patients per month (Author, submitted article). At the time of the study, the staff at the MHC consisted of four health workers: one psychiatric clinical officer, one psychiatric nurse, and two nursing aides.

### Sampling Strategy

Our study focused on caregivers, defined as individuals who, without being paid, “look after a family member, partner or friend who needs help because of their illness, frailty, disability, a mental health problem or an addiction, and cannot cope without their support” (38). As part of the larger study, caregivers were purposively sampled among adults from the outpatient waiting area of the MHC in Iganga district hospital, who were either: (i) accompanying a patient attending the MHC, or (ii) attending the MHC on a patient's behalf. Those who consented to the larger study, first took part in an MHC consultation observation and an exit-poll survey on patient-centred care, and consultation satisfaction outcomes, respectively (Author, submitted article). From this larger set of participants, caregivers who were residents of Iganga district, were further sampled at the end of the exit-poll survey to be part of this qualitative enquiry. A total of 27 caregivers agreed to be part of the qualitative study. Those who verbally consented to be part of this qualitative study were asked to provide their contact details for future follow-up for an in-depth interview at a time and place of their choosing.

### Data Collection

In-depth interviews were used for data collection, as a means to explore caregiver help-seeking itineraries that had been chosen on behalf of the MNS patients living in their care. Based on the aims of this study and the larger mixed-methods assessment of Iganga district hospital's mental health clinic (MHC) and its visiting patients and caregivers, a tentative interview guide was developed. The interview guide details can be found in the Supplementary File. The interview guide was an iterative document, flexible and amenable to change based on the responses of interviewees.

A total of 27 caregiver in-depth interviews were conducted. Prior to the interview itself, participants were given an information sheet in Lusoga and provided written informed consent. The interviews were conducted by a member of the research team, who was from Iganga and spoke the local language of Lusoga, and she has extensive experience using qualitative research methods for health-related research in this. Each interview lasted for approximately between 45 min−1 h and was conducted in the local language of Lusoga. Most interview locations were the housing compounds of the interviewees, or the fields on which they farmed at a time convenient to the interviewee. All interviews were digitally audio-recorded, transcribed verbatim, and translated into English by the interviewer.

### Data Analysis

Data analysis involved retroductive analysis, which uses both inductive and deductive coding processes. The retroductive process allowed for a dialogue between both emergent data as well as theory. Thus, analysis followed an emergent theory design based on data emerging from the interviews, the analysis of which was facilitated by the use of the PASS model.

The PASS-model was developed by the Partners for Applied Social Sciences and is based on anthropological theory (33). The model facilitated the analysis of a broad array of emergent elements that guided our analysis of caregiver help-seeking itineraries and patients' access to care, as well as exploring more systemic and structural factors at play. The model organizes relevant factors into four main categories: (i) explanatory models around illness perception; (ii) decision-making and its linkage to social values; (iii) access to care and resource seeking; and lastly, (iv) medical pluralism. Adding further support to these factors and helping build the context of help-seeking processes is the inclusion of context-specific worldviews, local social structures and cultural values, and the formal and informal health systems at play.

The process of data analysis began in fact during data collection. At the end of each interview day, daily debriefing sessions were held between the qualitative interviewer and NDPS. This in-field analysis allowed for an iterative and reflexive interview process in regard to thematically exploring key concerns and emergent themes as well as reaching data saturation. Field analysis also led to the creation of a data-driven codebook related to emergent thematic concerns arising from the narratives, such as personal motivators to provide care and the emergence of intra-household variations in pathways to care for multiple patients within the same household.

Once data collection was completed, all 27 interviews were analyzed using this retroductive process. This led to the identification of four households (i.e., four interviews) of special interest due to having multiple people within the same household who required MNS-related health care, but whose caregivers reported intra-household variations in the chosen pathways to care for each person. It should be noted that while these four cases were analyzed in further detail to understand and explore the intra-household variations, overall analysis from all 27 interviews is the basis of the presented results. The four households of special interest are indicative of the general themes found across all 27 interviews, with the difference of having multiple members within the same household with an MNS disorder. The process of analysis was regularly checked and discussed with remaining authors to ensure consistency in the findings. NVivo 11 was used to facilitate the storage, analyses, and further coding process of all transcripts.

### Ethical Considerations

Adult caregivers were informed about the study and provided initial verbal consent to be part of the qualitative study. Under-aged caregivers were not included in the study. The MHC patients themselves were not participants of this study. Prior to the interview itself, all participants were given an information sheet in Lusoga, and provided written informed consent for their participation in the interviews.

The study protocol, along with informed consent forms and tentative interview guides (including Lusoga translations), received ethical approvals from the Institutional Review Boards of the Institute of Tropical Medicine in Antwerp, Belgium; Makerere University, School of Public Health in Kampala, Uganda; and the Ugandan National Council for Science and Technology.

## Results

### Participant Demographics

A total of 27 in-depth interviews were conducted with the caregivers of MHC patients across Iganga district. The gender and age details of all participant caregivers and their related patients can be found in Table 1.

**Table 1:** Gender and age details of participant caregivers and related patients.

	**Caregivers (*****N*** **=** **27)**		**Patients (*****N*** **=** **27)**	
Female	19	70%	12	44%
Male	8	30%	15	56%
Age range	18–65	–	1–40	–
Median age	40	–	16	–

The majority of patients were children and adolescents: 19% were under age five, while 48% were between 6 and 17 years of age. The remaining third of patients were adults above the age of 18. The majority of caregivers were related to the patient (93%), female (70%), had a median age of 40, married (78%), and had primary school level of education (67%). The median household size was six persons. The majority of caregivers and the patients were Muslim, followed by Protestant, as well as being of the Busoga tribe.

### Help-Seeking Narratives

During analysis, intra-household variations in pathways to care for epilepsy, and other mental disorders were identified in four of the participant caregivers. As such, these four households were further selected for presentation as case studies and are showcased in this section.

Vignette 1 **Financial Difficulties**

According to Sheila, her daughter Agnes (F, 18 years) was 16 years when she began beating up people and abusing neighbors. Initially the family sought traditional help for a while, but Sheila still doesn't know what caused Agnes' illness—“*Some people may say that they bewitched me, and some say that they put witchcraft in my child's books. We thought maybe her friends did something because she was in a private school and we thought that maybe she ate something*.”

A friend told the family about “*a doctor who advertises himself on radio station in [neighboring district]. who operates on the human skull and he is so famous*.” But this was not followed up due to continual conflicts with the neighbors who were complaining about Agnes' abusive behaviors and “*thought that the child was pretending*.” The family had to resolve the dispute by going to the village local councilman. It was only this action which prompted Sheila, to request Agnes' father to finally take Agnes to a health facility. That first time the health workers thought it was diabetes; another time they thought it was typhoid.

Finally, a friend with an ailing elderly mother informed Sheila about the MHC, to which they had been going for approximately the last 1 year (at the time of the study). Sheila doesn't like one of the health workers who works there and thinks they should be changed. However, she does like another health worker who “*explains properly and tells you what is required and tells you what to do*”; although till date she hasn't yet once taken Agnes to have a face to face appointment at the MHC. Recently, her husband is getting tired of dealing with Agnes, but Sheila says she is still motivated to care for Agnes because she is the first-born child, and “*likes studying, is intelligent and the kind of child who respects her parents*.”

At the end of the interview, when we prompted to check if there was anyone else in the family who is ill or suffers from anything similar, Sheila hesitantly informs us of her son, Joseph (M, age unknown). Joseph started “*convulsing at age 6 month, and whenever he would convulse, he would become anemic*,” so for him they usually go to health centers for anemia treatment. At one point, his convulsing was thought to be (severe) malaria, for which they then sought malaria treatment. When we probe Sheila if she's ever taken Joseph to the MHC, she says “*I have thought of it… but because of lack of money, I haven't, because I thought [the girl] would get well soon so that I take [the boy], but what has brought up the delay is [the girl] not getting well*.”

Below we present four vignettes; each from the perspective of the caregivers of the four case presentations. The vignettes consist of narrative summaries of the interviews, including key quotes from the caregivers. They reflect varied help-seeking behaviors, chosen pathways to care and rationalizations made by the caregiver for the two patients who lived within each of their households. **Vignette 1** showcases the role of financial difficulties in ensuring care for both patients within the same household. **Vignette 2** highlights the fear of the label of epilepsy, a highly stigmatized illness in this context, and its effect on help-seeking. **Vignette 3** underscores the importance of good quality interpersonal care provision and the impact it can have on a caregiver. Finally, **vignette 4** calls attention to the influence of religion on help-seeking trajectories. To protect the identity of the participants, all names used within the vignettes are pseudonyms.

Vignette 2 **A Fear of “Epilepsy”**

The caregiver's name is Namaganda, mother to six children (two boys and four girls); her 4th born child, Esther (F, 15 years) is the one for whom Namaganda visits the MHC. Namaganda starts by describing how initially she refused to believe Esther's friends when “*they told me that my daughter could have become mad, and I said no it can't happen*.” Even when she saw first-hand that Esther “*had fallen on the ground and her eyes couldn't blink, I thought that maybe she has become dizzy.”* But later, Namaganda calls one of her sons to ask him that “*Is this thing also attacking Esther, because Mariam got better*?” That was when we first got to know that her eldest daughter and first born, Mariam (F, 20 years), also suffers from something similar.

After this episode, Esther was initially taken to a traditional healer who said, “*these children were bewitched using a grave of a person who had epilepsy so it's that thing following up these children*.” They sought traditional care for her for about 5 months, but there was no change in Esther's condition. In May 2015 Namaganda heard from a community member that there were “*certain whites in the district hospital who are treating the mental health disorder just like for your daughter*.” She enquired on it, but by then the “whites” had left [they were international researchers conducting a research study on epilepsy (39); instead, she came to know of the MHC and started attending there. However, soon after Namaganda stopped going to the MHC because “*when you hadn't gone with money, [one of the health workers] wouldn't give you medicine*.” She resorted back to traditional medicine, but by September 2016 thought “*the traditional world was just wasting my money and time, so I decided to go back to [the MHC] so that I get tablets, and whenever I don't get, I buy*.” By this time the previous health worker was gone, and since then a much better health worker is in charge whom she is very content with.

Mariam, Namaganda's eldest daughter and firstborn, was just 2 months old when she began to have convulsions, and the family tried to look for medications but not much treatment was found. It was only when Mariam was seven to 10 years of age, that the community members warned Namaganda to not “*neglect that daughter, because you may think it is convulsion, but when it is not convulsion, it may even result into epilepsy*.” The fear of it becoming epilepsy immediately prompted Namaganda and her husband to seek care at a neighboring health facility and start taking medication. However, soon the medication ran out and they didn't get her another dosage; eventually they resorted to traditional help—“*Fortunately she recovered but it remained on her eyes and could attack her eyes; maybe when she is talking and she keeps quiet*.”

We come to learn that Esther was first “attacked” when she was 1 year old: “*It could only attack half of her body, let us say maybe one leg, so when they brought medicine and tied on that leg, it would attack the other*.” She was taken to a health facility and treated for malaria with quinine; the mother now believes that it was the quinine that made her this way, because since then she can't speak properly. For Mariam, the mother believes it is linked to bewitchment, because she heard “*that there is someone in their clan who was of that kind*. I: With epilepsy? *Yes*.” Mariam does not attend the MHC.

### Factors Explaining Intra-household Variations in Pathways to Care

These four households are exemplary of the general themes found across all 27 interviews. Along with all other interviews, these four vignettes were retroductively analyzed, to thematically assess the most common factors that were likely to be decisive in choosing the appropriate pathway to care for each patient by their respective caregiver. While the PASS-model [Hausmann Muela et al. (31)] facilitated situating our findings, further analysis identified specific factors applicable particularly to this context and our findings. These identified factors were: (1). Contextual illness narratives ascribed for each patient; (2). Burden of caregiving and personal motivators to care; (3). Relevant social and cultural influences, including the therapy management group; (4). Socio-economic resources to provide continued care for that patient in the long term; and lastly, (5). Structural aspects of care—knowledge of available treatment options, access to, and quality of that care. In this section we discuss each of these identified factors and end this section with a reflection on epilepsy, as a socially and culturally stigmatized disorder in this context and how it shapes chosen pathways to care.

Vignette 3 **Quality of Care and Comfort**

Noori, the caregiver in this household, is the mother to Ali (M, 16), and the grandmother to Jamila (F, age unknown). Ali's illness began when he was around 3 years of age, in which “*all of a sudden, he started shaking and he fell down., and whenever he fell down, he would defecate and urinate himself*.” They initially went to the multiple traditional healers for 7 years until he was about 10 years old, at which point they stopped because “*they took my goats but he didn't heal. People told me that leave traditional medicine and go to the health facility*.” For several years thereafter, they received tablets from several nearby health facilities scattered in neighboring villages, but the travel and medication costs were all out-of-pocket.

A few years ago, they came across a traveling doctor who would visit their home and provide medication there, but “*after some time, that doctor stopped coming and we no longer saw him, so someone told us that there are free tablets at [the MHC]*.” Personally, she doesn't know what has caused Ali's illness, but that other people say “*that he has cerebral malaria and traditional healers say that it is cultural and family issues. Because two children can't fall ill from the same family*.”

Jamila is the Noori's granddaughter, and according to the Noori, “*malaria attacked her, and she started convulsing*” when she was around one and a half years old. For five and half years, until she was seven years of age, they sought traditional help for Jamila's condition. However, the caregiver worries that Jamila is not recovering as well as Ali. Noori states, “*Jamila doesn't realize when it's going to attack her, you find her suddenly down. [while] the change is there but very little because she convulses so much still. when it attacks Ali, it doesn't repeat*.”

Noori's experience at the MHC has been very positive, she receives medication free of charge compared to the past, and she finds that one health worker there “*talks to me as if I am his mother. He really shows concern and tells me to always pick the tablets and comforts me*.” She tries her level best to take care of the children, but wishes that the community wouldn't abuse the children—“*even these other children abuse the little ones, that look these ones have epilepsy, but I comfort them. Ali even cries when narrating to you after being abused*.”

Noori also worries about out-of-pocket expenses for medication since at times all the required medication is not available at the MHC. There were a time when she had to stop giving the children medication because she couldn't afford it, but then the children “*were too badly off and people told me to leave the boy and treat the girl*”; eventually she managed to resume both their treatments and improved their health.

#### Contextual Illness Narratives

In general, the specific context—namely, the time and place—of symptom onset, as well as perceived symptom severity and importance, played a role in the perceived causal illness etiology in the mind of the caregiver. This perceived causation shapes the contextual illness narrative created for the patient. The main symptoms that caregivers generally picked up on included: convulsions, “eye rolling,” being “lame” (often referring to physical disability in the legs and arms), speech impairment, and having inappropriate “abusive” behavior. Despite attending the MHC, the most common perceived etiologies for these symptoms were malaria (notably cerebral malaria); malaria treatment, particularly quinine; or epilepsy, which in this context is believed to be caused by bewitchment. Several caregivers also stated not to know causation. Others retained what the MHC health workers tell them and understand it as a brain disorder, linked to a lack of blood flow to the brain.

Vignette 4 **Religious Prompting**

We begin our interview enquiring about both the children who visit the MHC, and whom got the mental disorder first. According to their mother Prosper, it was the elder child Paul (M, 20), and it began immediately after his birth. They treated the boy for an infectious disease with blisters, soon followed by malaria treatment, but “*he couldn't even spend a week, maybe simple malaria attacks him, then he convulses, maybe you are sleeping, then he convulses*.” When aged six, they found a traveling doctor “*who could treat every illness. and gave some tablets like millet grains*” and the boy recovered slightly, being able to talk again and his lameness reduced for about 4 years. However, this doctor soon stopped visiting their home and Paul's treatment stopped. This was all before the birth of the second child, Charles (M, 10).

When Paul was 10, they heard on the radio about a mental health doctor in the neighboring district [at the regional hospital also having a mental health clinic], and after many efforts Prosper managed to find him who then gave some tablets for free. However, the neighboring district was too far and costly to travel to, and so Prosper looked in town for similar medication. She once even cheated an informal drug shop owner who “*didn't even know their price. I am the one who gave the price. and she accepted not knowing what loss she was making. but I failed to go back because she discovered the price*.” For a while after Prosper bought the medication at a private clinic. Eventually through the radio, Prosper came to hear that the medication had found its way to this MHC. This was back in 2014, and she was very happy to receive medication at the MHC, especially since there was one nurse who was kind to her.

Her second son, Charles' problem started when he was about 8 years old—Prosper began to notice that he had a lame arm and would knock many things over accidentally. But the mother admits there was a lot of delay in seeking care for him. It was only when 1 day while picking up Paul's medication at the MHC, that she found a ward “*where a certain lady was walking in a staggering way, so I went and asked what that ward was about, and the doctors said it was for lame people, so I decided to take Charles there because his hand was lame*.” After some treatment for the lame arm, Charles at some point got malaria, which led to convulsions. Prosper was “*worried so much that Charles too has also started to convulse and even thought of leaving him*.” But she kept silent about it: “*It was my secret and I didn't want [the doctor] to even know about it. But after some time, I was fed up, so I decided to go with him to the MHC*.” From 2015 onwards she has been visiting the MHC for both children.

Wrapping up the interview, we probe one last time about traditional medicine, to which the mother says “*The truth is that I would have tried but my religion doesn't allow such, but when others are conversing I hear what they have sold, goats, etc., and I thank God that he saved me from that, that I only use tablets from the hospital*.”

As was the case for Vignettes 1 and 2, even if caregivers had some awareness of a biomedical causation, knowledge of available MNS services, as well as accessing the MHC for one patient, they did not always immediately seek the same type of care for the second person within the household. This discrepancy in help-seeking and caregiver decision-making was linked to the differences in perceived causal links and timing of illness onset for each patient, limited/lack of knowledge on MNS disorders, as well as possible denial and rejection of a stigmatizing situation—namely, the fear of epilepsy.

#### Burden of Caregiving, and Personal Motivators to Care

The burden of care primarily fell on a female member of the family, usually the mother, but sometimes also the grandmother or elder sister. Fathers were frequently recalled as becoming easily fed up with the patients, who due to the chronic nature of their illness require significant time, attention, and financial resources spent on them. In Vignette 4, we see that the caregiver became so worried with the second child's illness onset, that she contemplated abandoning the child and even kept his symptoms a secret from the MHC health provider initially; it was only when she grew too exhausted from managing the situation that she finally also sought care for the second child at the MHC. Thus, the emotional capacity of caregivers was an important individual level factor affecting caregiver decision-making. Other personal motivators for the caregivers to continue taking care of their family members included the belief in God and fate; as well as the “goodness” of the patient/child and them deserving a better life.

#### Socio-Cultural Norms and the Therapy Management Group

Caregiver decision-making was influenced by socio-cultural notions related to the lay knowledge and meanings of illness symptoms. Caregivers were influenced by the perception of individuals around them, in particular the therapy management group—namely, those individuals close to the patient who partook in some manner of joint caregiving responsibilities. In some cases, the caregiver's contextual illness narratives were linked specifically to the patients' therapy management group members like the father or grandparents of the patient, while for other caregivers it was associated with the perceptions of surrounding community members, such as neighbors or friends.

These interactions with other members of society often shaped and reconsolidated the illness narrative in the mind of the caregiver. If the illness was highly stigmatized by the therapy management group and/or the larger community, such as was the case for epilepsy, this stigma would impact the caregivers chosen pathway to care for their patients. As such, the resultant contextual illness narratives were often a combination of the observed symptoms, their perceived etiologies, and socio-cultural norms and influence.

#### Socio-Economic Aspects of Care

The data also highlights how the cost of therapeutic care was a significant barrier to care being sought. Out-of-pocket expenditures for any form of treatment, whether it is traditional healing, neuropsychiatric medication, or even the travel costs associated with visits to a health facility, were important considerations in caregivers' foregoing one treatment option in favor of another lesser expensive option, or conversely returning to a previously forsaken option. The same choice—in how to *best* spend limited finances—extends to caregivers choosing *whom* to spend their money on treatment for when they have multiple persons to care for. In Vignette 1, the caregiver claims that the second child does not receive biomedical care at the MHC, due to insufficient funds to treat two patients simultaneously.

#### Structural Aspects of Care

Many of the caregivers in this study came to know of the MHCs existence through long-winded means—thus, knowledge of the MHC's existence and it being an option for treatment was a deciding factor in caregivers' decision-making. When caregivers were aware of the MHC and its available care provisions, it was of interest to note the importance of receiving *good* quality of care from the MHC—this was especially notable in Vignette 3. Having positive interpersonal relationships with health workers at the MHC was pertinent for caregivers. Perceived good quality care provision played a role in ensuring caregiver satisfaction and consistent use of the chosen therapeutic option. This was especially relevant in decision-making processes for illnesses that are otherwise stigmatized by the rest of society—it is already hard enough to take care of a patient with an MNS disorder, without having the additional burden of encountering rude or abusive care providers.

#### Epilepsy: A Socially and Culturally Stigmatized Disorder

Within this context epilepsy (like severe mental disorders) is believed to be a *clan illness*, and is highly stigmatized since it is socio-culturally linked to bewitchment, spirit possession, and familial curses. This stigma is especially notable in cases where the patients are discriminated against and bullied by community members for having epilepsy like in Vignette 3, or when traditional healers claim the hereditary link can only be due to bewitchment like in Vignette 2. For contextual illness narratives that are linked to epilepsy, caregivers almost always resort in traditional help at some point in the overall help-seeking trajectory.

This stigma can also extend to the biomedical treatment available for epilepsy at public health facilities, including the MHC. For instance, in the case of Vignette 3, it was the fear of convulsions turning into the socio-culturally stigmatized epilepsy, which forced the caregiver to seeking biomedical care in the case of the second child. This implies that convulsions (which may be a familiar event for children under five and linked to fever, malaria or meningitis) by themselves are not necessarily perceived as being equivalent to the cultural form of epilepsy. By this logic, it is possible that in the mind of the caregiver, convulsions can be treated biomedically, while epilepsy cannot.

Knowing these contextual understandings, the health workers at the MHC have come up with an ingenious grounded solution to combat any associated stigma with epilepsy—they simply do not use the word “epilepsy,” neither verbally nor written in their diagnoses of patients. Rather they refer to the specific type of seizure by its medical name such as “grand mal seizures” or “temporal lobe seizures.” The caregivers, who are lay members of society with limited medical knowledge, are generally content to have this medical terminology—which they do not know the meaning of—written in the patient medical booklets. They continue to seek the appropriate biomedical care for it at the MHC, without ever linking it to the socio-culturally stigmatized illness of epilepsy.

## Discussion

**Figure 1:**
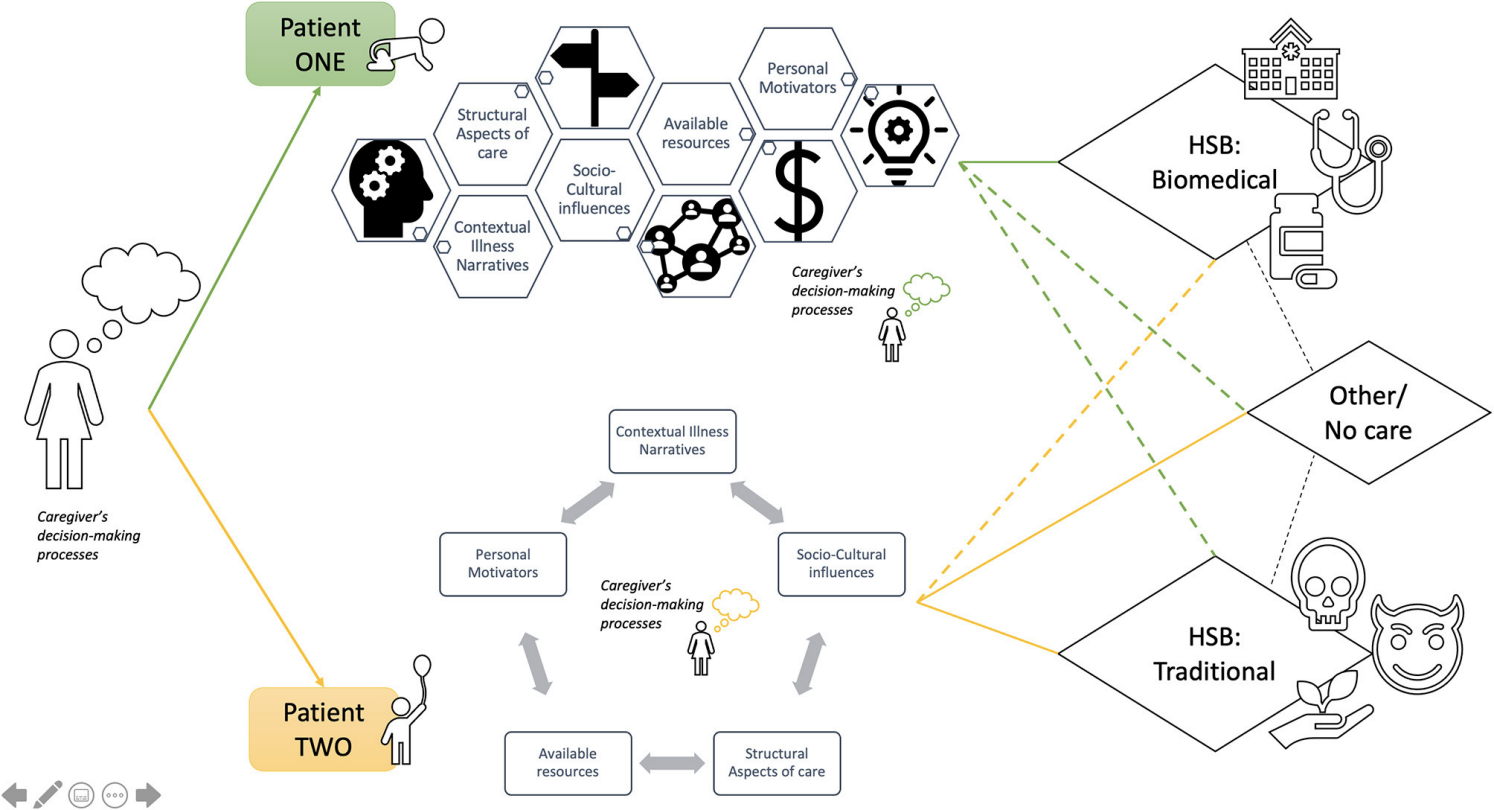
Illustration of possible intra-household variations in chosen pathways to care by a caregiver of two persons with epilepsy and mental disorders living within the same household.

Our data showcase that across all interviews caregivers' initial chosen pathways to care for individual patients were based on a multiplicity of factors that converge to influence their differential decision-making processes. The interplay of these factors in caregiver decision-making processes were different from one patient to another. The resultant selected pathways to care were flexible, variable and uncertain; in some cases, even amongst patients within the same household by the same caregiver. Figure 1 is an illustration of various decision-making approaches that may occur for different patients by the one same caregiver in cases of intra-household variation. The figure can be viewed as a theoretical construction of how the complexity of intra-household variation in chosen pathways to care, by caregivers of persons with MNS disorders, may play out within this context.

Differences in care seeking pathways depend on the caregiver's perceived explanatory contextual illness narratives around the specific disorder for each patient. This is similar to other documented instances for MNS disorders across Uganda (13, 30, 40, 41). However, in this study of particular interest was how caregivers attribute a given contextual illness narrative to one patient over another, in some cases even within the same household. We hypothesize that if the caregiver ascribes a different contextual illness narrative to the second person within the household, it could naturally then lead to a different chosen pathway to care than the first patient.

Patient preferences and autonomous decision-making for their own health should also be taken into account and advocated for respectively, especially in this context (42). However, considering the burden of MNS disorders and depending on the ascribed contextual illness narrative for each patient, caregiver seek out appropriate care pathways for that patient. As shown in previous studies on mental disorders in this context, traditional medicine and religious care can often be either a first recourse to help or a secondary option after biomedical care fails (13, 16, 21, 40). These help-seeking itineraries are often multi-sectoral and can be both simultaneous or sequential in nature (30).

The consequence of intra-household variations is that one patient may receive prioritization over the other, in terms of who has the available resources spent on them, and who receives what type of care, especially in resource-constrained settings such as these. It is important to recognize that personal motivators can, both unintentionally or intentionally, influence a caregiver in favoring the recovery of one child over another. This intra-household prioritization of access to preventive care or treatment is reported to be a neglected area of enquiry within health-seeking behavioral studies (33).

This leads us to the notion of social vulnerability (43) and more specifically patient vulnerability. Patient vulnerability in being prioritized or not when receiving care may be influenced by factors such as higher parity of the mother (in case she is the main caregiver), or the child's birth order or gender. There is a dearth of research in this particular line of enquiry; however, one study in a high-income country indicates that girls are much less likely to obtain needed treatment for particular mental disorders than are boys, while middle birth order children are less likely to receive mental health care and treatment than are oldest, youngest or only children (44).

Nonetheless, it is also important to emphasize that these processes of prioritization are (intrinsically) dynamic in time and space; they have their own rationality and they are not necessarily problematic. They can also be viewed “positively,” as an indication of flexibility and adaptability of the caregivers, considering the unique character of every patient and the variability in resources available. Further research is required on these social factors, the prioritization and selection of care, and their impact on patient vulnerability by caregivers of persons living with MNS disorders in this and other low-resource settings.

In a few cases, the caregiver whom we met at the MHC was the father of the patient. The father's role was primarily to provide financial support toward chosen treatments, which is similar to findings from another recent Ugandan study on epilepsy (14). One study in the USA reports that having larger numbers of adults within a household helps in children receiving treatment, by possibly reducing the burden of care and impact of resource-constraints; while the presence of a father inhibits the likelihood of a child receiving care (44). However, it remains to be seen to what extent this would be valid in sub-Saharan African contexts, where the father is often the economic provider for a family.

Our study also highlighted a pronounced caregiver fatigue in relation to their duties for patients with chronic MNS disorders, like that of epilepsy. This fatigue in providing care can be especially observed among primary caregivers, who face the largest burden of care, and is even more likely when a second child also falls chronically ill in the same manner (14). The data indicates there was a withholding of information and delay in seeking help in some instances. This can also be viewed as some form of control and/or a coping mechanism in an otherwise chaotic and exhaustion-inducing scenario. It also highlights that there still exists an unmet need for the recognition of relatives and families as “caregiving experts,” and as genuine partners in patients' pathways to care and health trajectories, as well as in biomedical and psychiatric practice and research (45).

In a system where the caregivers of people with MNS disorders remain undervalued and their contributions to the formal and informal health system outweigh the recognition they actually receive (46), these findings highlight the importance of acknowledging the role that intrinsic and extrinsic motivators play in how, why and to whom caregivers ensure and provide MNS care for, especially in low-resource circumstances. Strategies focusing on caregiver attitudes and information sharing have been found to translate into more positive outcomes, while encounters with health professionals can be perceived as frustrating to caregivers (47). Increased perceived autonomy supportiveness of caregivers, alongside that of the patients, may motivationally impact caregivers in their consequent care-giving behaviors through adjustments in their emotional and cognitive capacities (47). It has also been found that changes in the process and content of family psycho-education—reflecting the specific social, cultural and gendered realities—worked best for postpartum psychosis in central Uganda (28). These strategies may be of relevance for this specific context, where the improved emotional and social support of caregivers, as well as the inclusion of the therapy management group and group care may yield to better patient outcomes through joint decision-making processes.

In the creation of a contextual illness narrative (which can be thought of as an automatic perceptual and cognitive reaction to understanding a given health situation), the caregiver is also placing a label or “tag” in defining the problem. In labeling theory, these attitudes and actions toward stereotyping are linked to stigmatizing outcomes (48). Thus, the label of “being bewitched,” being “mentally ill,” or having “epilepsy” are additional factors that may influence any actions taken toward help-seeking. In this specific context these labels do not simply affect the patient, but by association can also touch upon the larger family and extended relatives' group. Referred to as courtesy stigma or social stigma, this process of stigmatizing a phenomenon through its labels has been previously reported cross-culturally, as well within the Ugandan context, especially as linked to MNS disorders and, in particular, to epilepsy (49–51).

The reflections on epilepsy as a socially and culturally stigmatized disorder highlight how this stigma was locally managed by MHC staff in efforts to provide continued biomedical care. While, the existence of this stigma is largely supported by the literature (52, 53), these grass-roots tactics are important to note, as there is a substantial gap in knowledge, and interventional research to reduce MNS-related stigma and discrimination, especially in low-income settings (54). This is particularly relevant in Uganda, where estimated country-level prevalence of epilepsy has been reported to be as high as 10.3 per 1,000 people in eastern regions of the country (39).

While the ability to pay for health care is variable, financial facilitators or barriers are likely to remain influencing factors in caregiver decision-making processes especially in resource constrained settings. From a socio-economic perspective, out-of-pocket expenses are known to contribute directly to pluralistic therapeutic itineraries (15). The implications for the MHC are that access to free psychotropic medication for the patients acts as an effective motivator for the caregivers in accessing biomedical care for the patients. As such, the MHC can be a key therapeutic choice especially once caregivers see active and visible improvements in the patient's condition once stabilized on them through sustained pharmacological treatment. However, for patients who have only recently started pharmacological treatment and show little or no immediate recovery, the doubt about the benefits of biomedical care in combination with out-of-pocket payments, may likely revert caregivers toward other pathways of care or leave patients without care.

Thus, due to the chronic nature of epilepsy or other mental disorders that require long term pharmacological treatment to prevent relapse, unavailability of psychotropic medication at the MHC, drug-stockouts, and the resultant out-of-pocket medication purchases remain significant barriers to ensure sustained biomedical care (13, 14). There is likely a constant cost-benefit analysis that caregivers are performing in their decision-making processes, sometimes even at the expense of both patients' health. Poverty and associated vulnerabilities indeed shape patient health outcomes, via the pathways to care, or lack of, that are ultimately chosen by the caregivers.

This link between poverty and mental health outcomes is well documented (49, 55). In this study, with financial barriers and out-of-pocket expenses being a significant barrier to biomedical care—barriers that further encourage pluralistic health seeking behaviors—it is important to recognize the need for ensuring universal health care for MNS disorders (56). Bringing MNS concerns to the forefront for reflection and prioritization in the Ugandan public health system would encourage the country toward the creation of a holistic, high-quality, patient-centered health system as advocated for by the global health community (57, 58).

Finally, it is crucial for MNS patients and their caregivers to have knowledge and awareness of what MNS services and provisions exist in reality and what is accessible to them, especially for stigmatized conditions like epilepsy (59). Aside from the social and economic vulnerabilities of MNS disorders, the widespread stigma—attached to MNS patients, their caregivers and mental health specialists alike—acts as deeply ingrained social, structural, and even institutional barriers toward ensuring equitable MNS care (45, 52, 53, 60, 61). The equitable coverage of quality MNS care is essential if we are to ensure that patients and their caregivers have access to safe, effective, timely, efficient, equitable, and patient-centered care (56). The right to health, and especially that of humane care, should be a basic underpinning in providing treatment for MNS disorders, regardless of context, and resource constraints (7).

## Conclusion

This study showcases the variety of ways in which mental, neurological and substance-use disorders, in particular that of epilepsy, are conceptualized by the caregivers of patients with MNS disorders in rural eastern Uganda. Results show that associated help-seeking itineraries for MNS disorders are largely pluralistic; oftentimes combining and alternating between various pathways to care. In unraveling *how* and *why* intra-household variations to pathways of care for MNS disorders occur, we highlight that these variations are likely a result of the complex interplay of individual, social, financial and structural factors, especially in resource-constrained settings. Finally, if the equitable coverage of quality MNS care and the presence of high quality mental health systems of care is to be achieved in the long run, then further research and prioritization of MNS health care in global health policy, and practice is of urgent essence.

## Limitations

This study was a one-time qualitative exploration from the perspectives of the caregivers. There is no longitudinal data on how stable caregiver contextual illness narratives are or how they influence one another over time. There is also the question of reliability of lay conceptualizations and diagnoses of MNS disorders in the context of Iganga. The crystallization and stability over time of these caregiver contextual illness narratives, their linked help-seeking trajectories for MNS patients, and the reliability of illness diagnosis would be of interest for further study. Additionally, future study on patient perspectives (for those having capacity), their preferences, and autonomous health-related decision-making would be also of interest to complement caregiver perspectives in a setting such as this.

## Data Availability Statement

The data supporting the findings of this study/publication are retained at the Institute of Tropical Medicine, Antwerp and are not openly accessible due to ethical and privacy concerns. Pseudonymised data sets can however be made available after approval of written requests to the Institute of Tropical Medicine at ITMresearchdataaccess@itg.be/.

## Ethics Statement

The studies involving human participants were reviewed and approved by Institutional Review Boards of: Institute of Tropical Medicine in Antwerp, Belgium; Makerere University, School of Public Health in Kampala, Uganda; and Ugandan National Council for Science and Technology. All participants provided their written informed consent to participate in this study.

## Author Contributions

NDPS and BC designed the study. NDPS collected and analyzed all data. JMR and KPG provided critical analyses of results. NDPS drafted the manuscript. All authors critically reviewed manuscript drafts and have consented to this manuscript.

## Conflict of Interest

The authors declare that the research was conducted in the absence of any commercial or financial relationships that could be construed as a potential conflict of interest.

## Publisher's Note

All claims expressed in this article are solely those of the authors and do not necessarily represent those of their affiliated organizations, or those of the publisher, the editors and the reviewers. Any product that may be evaluated in this article, or claim that may be made by its manufacturer, is not guaranteed or endorsed by the publisher.
